# Bioaccumulation, Bioindication and Health Risk Assessment of Heavy Metals in Cape Horse Mackerel (*Trachurus trachurus*) and Slinger Seabream (*Chrysoblephus puniceus*) in the Durban Basin and Cape Vidal, South Africa

**DOI:** 10.1007/s00244-023-01028-8

**Published:** 2023-08-17

**Authors:** Sanjeev Debipersadh, Henry Joseph Oduor Ogola, Kevin Mearns, Ramganesh Selvarajan

**Affiliations:** 1 Department of Environmental Science, University of South Africa- Florida Campus, Roodepoort, 1709 South Africa; 2 School of Food and Agricultural Sciences, Jaramogi Oginga Odinga University of Science and Technology, Bondo, Kenya; 3 Laboratory of Extraterrestrial Ocean Systems (LEOS), Institute of Deep-Sea Science and Engineering, Chinese Academy of Sciences, Sanya, People’s Republic of China

## Abstract

The bioaccumulation of heavy metals (HMs) in marine fish is a growing global concern due to potential human health risks. The study analyzed HM in the muscle tissue, gills, and gut of adult male and female cape horse mackerel and slinger seabream caught in the polluted Durban Basin and pristine Cape Vidal from April 2018 to February 2019. Results revealed interspecific, spatial, and organ-specific variability in HM levels. In the Durban Basin, slinger seabream had bioaccumulation (in mg/kg) of As (2.3 ± 0.2*)*, Cr (2.6 ± 0.2), Ni (2.0 ± 0.1), and Pb (4.1 ± 0.3) while cape horse mackerel had Ni (1.6 ± 0.2), Pb (4.7 ± 0.6), and Zn (52 ± 3.01) exceeding World Health Organization (WHO) regulatory limits. Metal pollution index (MPI) values were also higher in Durban Basin (> 5.13) than Cape Vidal (< 3.32) for both species’ muscles. Liver and gills of slinger seabream and gut of cape horse mackerel exhibited higher HM accumulation patterns proportionate to the environmental concentrations, indicating the bioindicative potential of HM pollution by the two species. Risk assessment indicated that both fish species had target hazard quotient > 1 for Cr, and target cancer risk < 10^–4^ for Pb, implying significant potential non-carcinogenic and carcinogenic health risks associated with fish consumption from the Durban Basin. The study recommends daily consumption limits of 16 g/day for slinger seabream and 14 g/day for cape horse mackerel to ensure health safety. The findings contribute to the understanding of HM pollution in the Durban Basin and provide important information for decision-makers and policymakers in developing effective strategies to mitigate and manage HM contamination in fish populations.

Globally, human activities have a direct impact on the quality of surface water in various ecosystems, including marine environments. The entry of anthropogenic pollutants into coastal areas, including marine environments, is primarily caused by factors, such as economic development, industrialization, and urbanization (Liu et al. 2007; Stark et al. 2014; Vetrimurugan et al. 2019). The Durban Basin, which is home to South Africa's largest and busiest port in terms of cargo values, as well as an economic hub that supports numerous manufacturing, petrochemical, sea trade, and transport industries (Goble et al. 2014), has experienced the impact of rapid industrial and urban development, coupled with associated ancillary activities, on heavy metal (HMs) pollution of the KwaZulu-Natal (KZN) coastal environment. This issue has been an area of increased research interest (Moloney et al. 2013; Mzimela et al. 2014; Okoro et al. 2016; Vetrimurugan et al. 2019).

Fish are one of the main sources of protein for human diet worldwide, but they are also known as being the largest bioaccumulators of HMs due to their position at the top of the food web/food chain in both freshwater (Thompson et al. 2012) and marine ecosystems (Hernández-Almaraz et al. 2014; Vilches et al. 2019). High content of HMs, including both transitional metals and metalloids, can have potential adverse effects on human health and the environment. While transitional metals such as Co, Fe, Cu, Zn, Ni, and Mn are generally essential in low concentrations, they can be toxic at high concentrations (Santamaria 2008; de Romaña et al. 2011; Nriagu 2011; Jaishankar et al. 2014; Bosch et al. 2016). In contrast, non-biogenic metals and metalloids such as As, Cd, Pb, Hg, Se, and Cr and their organometals such alkylated lead, tributyl tin and methylmercury, are extremely toxic even at very low concentrations (Mann et al. 2011; Bosch et al. 2016; Ali and Khan 2018). Ecologically, these non-biogenic HMs are generally persistent in the environment due to their non-biodegradable and recalcitrant nature and can undergo trophic transfer and bioaccumulation along an aquatic food web, posing a significant risk to both the environment and human health. Fish is an important source of food for the general human population along the South African coastline, and there are several reports on the correlation between human health hazards and the consumption of contaminated fish and other aquatic foods (Gu et al. 2015; Bosch et al. 2016; Yi et al. 2017), with some studies proposing using fish species as potential pollution indicators and for monitoring toxic HMs accumulation in the marine environment (Canli & Atli 2003; El-Moselhy et al. 2014; Jayaprakash et al. 2015). However, it important to note that to select a bioindicator species to assess human health effects, it should meet certain criteria such as being commonly consumed in the area, widely distributed geographically, having the potential to accumulate high metal concentrations, and having adequate tissue mass for residue analysis (Cunningham et al. 2019).

To understand the potential risk of HMs in fish to consumers, it is essential to obtain contemporary data on the levels of toxic HMs in a variety of fish species. Environmental conditions and biological factors might influence metal levels in fish from the Durban Basin, and there is a need to identify fish species of which consumption may be a threat to human health. To the best of our knowledge, there are limited studies that have been conducted on the relationship between HMs concentration and edible fishes in the Durban Basin, with anecdotal evidence reporting high accumulation of Al and Zn in six edible fish species (Debipersadh et al. 2018; Moodley et al. 2021). These studies indicated that the health risks associated with consuming these fish species were relatively low. Nevertheless, the applicability of these findings to the entire Durban Basin remains uncertain due to the confined range of sampling sites and the small sample size employed in the studies. As a follow-up study by Debipersadh et al. (2018), cape horse mackerel (*Trachurus trachurus*) *and* slinger seabream (*Chrysoblephus puniceus*) were sampled in the wider Durban Basin (from Umdloti to Isipingo) and compared against those from a pristine coastal ecosystem of Isimangaliso Wetland Park in Cape Vidal to gain further insight into possible HM contamination in the Durban Basin. This study aimed to investigate the distribution of HMs in different organs of two fish species from the polluted Durban Basin and the pristine coastal system, assess the human health risks associated with fish consumption, and explore the bioindicative potential of these fish species for monitoring HM accumulation in the Durban Basin.

## Materials and Methods

### Study Area Description

**Fig. 1:**
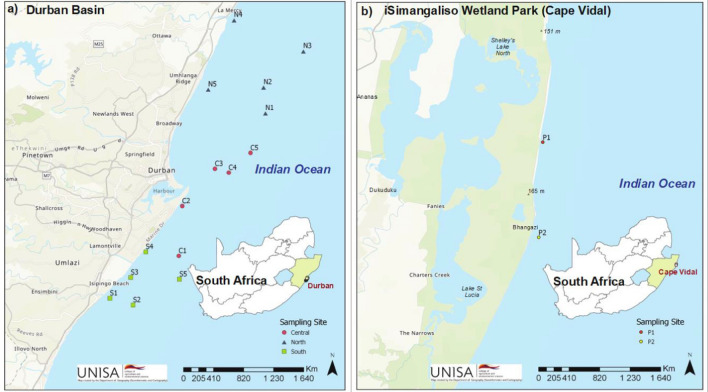
Map of the entire Durban Basin (**a**) and iSimangaliso Wetland Park (Cape Vidal) (**b**) showing the location of sampling points. Inset of each map shows the location of the study area in South Africa

The study aimed to compare the accumulation of HMs in fish from the Durban Basin, a region known for its industrial activities and domestic sewage treatment plants, with a pristine coastal system, the iSimangaliso Wetland Park, which is a protected World Heritage Site known for its biodiversity. The Durban Basin extends from Umdloti (29°39′ 0′′ S 31° 8′′ 0′′ E) in the north to Isipingo (30° 0′′ 0′′ S 30° 56′ 0′′ E) in the south, and encompasses areas with various industrial activities, including petrochemical, pulp and paper, beverage, textile, plastic, and motor vehicle industries. The “Central” region includes Durban harbor, a prominent shipping industry hub, and is serviced by an outfall pipeline that discharges a significant amount of sanitary wastewater into the ocean. The northern coastline of the Durban Basin, referred to as the “North,” includes the Umdloti and Umgeni rivers, which receive partially treated sewage wastes from adjacent wastewater treatment plants before discharging into the ocean. Fish samples of cape horse mackerel and slinger seabream were collected from five different sites in Durban South (S1-S5), Central (C1-C5), and North (N1-N5), as depicted in Fig. 1a.

The iSimangaliso Wetland Park (27° 40′ 36.8" S 32° 37′ 18.6" E) was chosen as a reference site, and samples of cape horse mackerel and slinger seabream were collected from two locations within the Cape Vidal Controlled Zone. The study received approval from both the Department of Environmental Affairs and iSimangaliso Wetland and Marine Park. Sampling was conducted in the Cape Vidal Controlled Zone and Catch and Release Zone, with P1 located approximately 4.5 km north of Cape Vidal and 2.5 km north of the Cape Vidal Lighthouse, and P2 located approximately 2.5 km south of the Cape Vidal Lighthouse, as illustrated in Fig. 1b.

### Sampling and Sample Preparation

Between April 2018 and February 2019, a total of 62 slinger seabream and 18 Cape horse mackerel were sampled from five different points in the Durban Basin, representing various regions. In addition, 12 fish samples, including six slinger seabream and six cape horse mackerel, were collected from the pristine Cape Vidal marine environment for comparison with pollution levels in the Durban Basin. The fish samples obtained were a mix of males and females. The mean length and body mass for slinger seabream were 303.9 ± 47.0 mm and 722.0 ± 3.7 g, respectively. For cape horse mackerel, the corresponding values were 202.4 ± 17.3 mm and 76.7 ± 0.1 g. The average depths at which the two fish species were caught were 40.3 ± 15.2 m and 22.3 ± 5.4 m deep for slinger seabream and cape horse mackerel, respectively.

The fish were caught using spearfishing and rod and reel with baited hooks in deep areas where spearfishing was not possible. After catching, the fish were immediately chilled on ice to maintain sample integrity. Due to their large size, muscle tissues, gills and liver samples of slinger seabream were collected onsite into separate sample bottles, with the remaining fish parts being discarded back into the ocean to serve as a food source for other fish. On the other hand, given the smaller size of the cape horse mackerel, we opted to collect whole fish samples in the field. Subsequently, in the laboratory, we carefully separated the muscle tissue, frame (fish skeleton including fish fins), and gut samples for further HM analysis. All fish samples for metal analysis were transported to the laboratory at 4 °C in a cooler box with ice for further analysis.

### Heavy Metal Analyses

To determine the total HM concentrations (Al, As, Cr, Cu, Mn, Ni, Pb, and Zn) in fish samples, the following steps were taken. First, the fish samples were prepared by drying them in a porcelain crucible to a constant mass at 105 °C for 24 h. The dried samples were then ashed at 600 °C for 6 h to produce an inorganic residue suitable for downstream analysis of the total amount of metals within the fish sample. To prevent the loss of metals, the ashing temperature was maintained at 600 °C. The resultant ash was milled to a fine particle size prior to digestion of 0.5 g with 30 mL of HCl and 10 mL of HNO_3_ on a hot plate maintained at 100 °C until all the sample was completely dissolved. The resulting solution samples were then evaporated to a volume of 5–10 mL followed by cooling to room temperature and mixing with 2.5 mL of an internal indium standard. The digestions were performed in triplicate for each sample.

The total HM concentrations were determined using inductively coupled plasma-optical emission spectrometry (Optima 8300® ICP-OES, Perkin Elmer Inc., Watham, MA, USA) according to the manufacturer's instructions and as described by Debipersadh et al. (2018). The limit of detection (LOD) for the instrument was 10 µg/g, and all analytical results were expressed as µg/g dry mass. Every ICP-OES measurement began with a calibration blank solution containing an indium internal standard, followed by running the eight calibration standards, then samples, and finally ending with the calibration blank. Sample analysis was only done when the calibration blank reading was below the instrument LOD. During the analysis, every element was analyzed in triplicate to ensure accuracy and precision.

The metal pollution index (MPI), a measure of overall contamination of fish tissues with HMs, was determined according to Eq. 1 described by Usero et al. (1997).

1 $$ {\text{MPI}} = (M_{1} \times M_{2} \times M_{3} \times \cdots \times M_{{\text{n}}} )^{{1/{\text{n}}}} $$

where M_1_ is the concentration of first metal, M_2_ is the concentration of second metal, M_3_ is the concentration of third metal, M_n_ is the concentration of “n” metal (mg/kg dry weight) in a certain tissue.

### Human Health Risk Assessment

Dietary exposure to HMs from cape horse mackerel and slinger seabream consumption by an adult was calculating estimated daily intake (EDI) according to Eq. 2 (Song et al. 2009):

2 $$\mathrm{EDI }=\frac{C \times D }{\mathrm{Aw}}$$

where C is the average concentration of HM in fish (mg kg^−1^); D represents the daily fish consumption rate of (g day^−1^). In South Africa, the average annual fish consumption per person in South Africa is estimated to be 6–8 kg (~ 21.92 g day^−1^) (Grünberger 2014); and Aw is the mean body mass of an adult (in kilograms). For calculation, the average body mass in Africa of 60.7 kg (Walpole et al. 2012) was used.

We used the target hazard quotient (THQ) proposed by the US Environmental Protection Agency (US.EPA 2009) to assess the potential risk to people who consume cape horse mackerel and slinger seabream due to trace elements present in these fish. The THQ provides an estimate of the non-carcinogenic risk level associated with exposure to pollutants, and it is calculated using the following Eq. 3:

3 $$\text{THQ} = \frac{{M}_{\mathrm{c}}\times \mathrm{IR}\times {10}^{-3}\times \mathrm{EF}\times \mathrm{ED}}{{R}_{\mathrm{f}}D\times \mathrm{BW}\times {\mathrm{AT}}_{\mathrm{n}}}$$

THQ is dimensionless value, and the variables in the equation are defined as follows: M_*c*_ is the mean metal concentration in the fish tissue (mg kg^−1^), IR is the ingestion rate (g day^−1^), *EF* is the exposure frequency (365 days year^−1^), ED is the exposure duration (64.2 years, South Africa the average life expectancy (Stats SA 2019), BW is the body weight (kg) (60.7 kg average body mass for African adult), RfD is the reference dose of the individual metal (mg/kg/day), and AT_n_ is the average life time exposure for non-carcinogens (365 days/year x ED). If the THQ value is greater than one (i.e., THQ > 1), it indicates that the exposed population is likely to experience adverse health effects. The probability of hazardous risk increases with the THQ value. To evaluate the risk associated with multiple HM present in fish, we calculated the total hazard index (HI) by summing the THQi values for each metal, as described by Eq. (4):

4 $$\mathrm{HI }= \sum_{i=1}^{n}\mathrm{THQ}i$$

THQi is the target hazard quotient for an individual metal, and n equals 8 in the present study, which corresponds to the number of metals analyzed.

Carcinogenic risk (TR) due to consumption of cape horse mackerel and slinger seabream was calculated according Eq. 5 according to USEPA (2005):

5 $$\mathrm{TR}=\frac{{M}_{\mathrm{c}}\times \mathrm{IR}\times {10}^{-3}\times {\mathrm{CPS}}_{\mathrm{o}}\times \mathrm{EF}\times \mathrm{ED}}{\mathrm{BW}\times {\mathrm{AT}}_{\mathrm{n}}}$$

where M_*C*_, IR, EF, ED, Bw and AT_n_ are already explained in Eq. 3. CPS_o_ is the carcinogenic potency slope, oral (mg/kg bw-day^−1^). Only the *TR* value to show the carcinogenic risk due to intake of As, Ni and Pb using the slope factor CPS_o_ as stipulated by USEPA (2005). The potential carcinogenic effects of metals (As, Ni and Pb) was further characterized by calculating the maximum allowable daily fish consumption limit (TR_lim_). TR_lim_ was determined by following Eq. 6:

6 $${\mathrm{TR}}_{\mathrm{lim}}=\frac{(\mathrm{ARL x BW})}{(C\mathrm{ x }{CPS}_{o})}$$

where, ARL is maximum acceptable risk level (10^–5^, unitless); BW is mean adult body mass in kg (60.7 kg for South African population); *C* is mean metal concentration in the different fish species; and CPS_o_ is the cancer slope factor (1.5, 1.7 and 0.009 mg/kg/day for As, Ni and Pb, respectively) (USEPA 2000). In addition, the allowable number of fish meals of a specified size that may be consumed over a given time period (TR_mm_) was also calculated using the following equation according USEPA (2000):

7 $${\mathrm{TR}}_{\mathrm{mm}}=\frac{({\mathrm{TR}}_{\mathrm{lim}}\mathrm{ x }{T}_{\mathrm{ap}})}{\mathrm{MS}}$$

where TR_mm_ is maximum allowable fish consumption rate (meals/month); *T*_ap_ is time averaging period (365.25 days/12 months = 30.44 days per month), MS is meal size (0.227 kg/day fish/meal for adults person (USEPA 2000).

### Statistical Analysis

The HM content of trace elements in cape horse mackerel and slinger seabream were compared to determine their differences, and site-specific means of each species were used for the comparison. Parametric one-way ANOVA and nonparametric Kruskal–Wallis and Mann–Whitney U tests were used for the analysis, and Tukey's HSD was applied for pairwise mean comparisons in the ANOVA post hoc test, when homogeneity of variance was established. The accumulation and partitioning of HMs in different tissues of cape horse mackerel and slinger seabream were compared within each sampling site using the parametric Student's t-test and nonparametric Mann–Whitney U test. To test the correlation between trace element concentrations in the fish, parametric Pearson's correlation test and nonparametric Spearman's rank correlation test were used as appropriate. In addition, principal component analysis (PCA) and hierarchical cluster analysis based on Bray–Curtis similarity were used to determine the correlation between heavy metals in fish tissue and to differentiate the samples' contamination status. All statistical analyses difference were conducted at a significance level of 0.05 using various R software packages (R Core Team 2021).

## Results and Discussion

### HMs Concentration in the Different Body Parts of the Two Fish Species

HM accumulation in fish can pose a significant risk to both aquatic animals and humans, and determining the concentration of HMs is the first step in evaluating contamination levels. In this study, ICP-OES analysis was employed to measure the concentration of HMs, including Al, As, Cr, Cu, Mn, Ni, Pb, and Zn, in 12 cape horse mackerel and 62 slinger seabream fish samples caught in the Durban Basin, located on the South African coastline of the Indian Ocean. Table 1 displays the mean concentrations of these HMs in various parts of the fish's body, such as muscles, gills, liver, gut, and frame, for the two fish species.

**Table 1:** Mean (± SE) comparison of heavy metal concentration (mg/kg dm) and metal pollution index (MPI) in different body parts of cape horse mackerel and slinger seabream in Durban Basin

	Cape horse mackerel (*Trachurus trachurus* L.)			Slinger seabream *(Chrysoblephus puniceus* L)		
Body mass (g)	76.7 ± 0.1			722 ± 3.7		
Total length (cm)	30.2 ± 7.3			304 ± 47.0		
Average depth caught (m)	22.3 ± 5.4			40 ± 15.2		

Lifestyle	Benthopelagic			Demersal		
Body parts	Muscle	Frame	Gut	Muscle	Gills	Liver
Al	20 ± 3.40*a*	11 ± 1.83*a*	41 ± 5.99b***	32 ± 2.71*a*	58 ± 4.85*b*	64 ± 5.81b***
As	1.0 ± 0.07*a*	2.0 ± 0.23*b*	1.7 ± 0.16ab***	1.7 ± 0.28*a*	3.6 ± 0.29*b*	1.7 ± 0.28a***
Cr	1.7 ± 0.25*a*	2.1 ± 0.12*ab*	2.4 ± 0.26b*	2.7 ± 0.25*a*	3.7 ± 0.30*b*	3.6 ± 0.32ab*
Cu	5.0 ± 0.54	3.5 ± 0.48	4.5 ± 0.58	3.6 ± 0.26*a*	3.8 ± 0.25*a*	4.9 ± 0.36b**
Mn	1.2 ± 0.11*a*	2.4 ± 0.18*b*	2.9 ± 0.26b*	3.2 ± 0.29*a*	11 ± 1.09*b*	3.1 ± 0.27a***
Ni	1.4 ± 0.20	1.8 ± 0.12	1.4 ± 0.09	1.9 ± 0.17*a*	3.1 ± 0.17*b*	3.4 ± 0.32b***
Pb	4.1 ± 0.51*a*	2.4 ± 0.26*b*	5.0 ± 0.61a**	4.0 ± 0.36	4.4 ± 0.45	5.1 ± 0.39
Zn	47 ± 3.02	54 ± 3.42	56 ± 4.89	26 ± 1.67*a*	55 ± 3.41*b*	58 ± 4.02b***
MPI	5.66	5.25	7.72	3.96	9.45	9.10

Mean values followed by different letters within a row are statistically different (ANOVA; Tukey’s HSD test, at *p* < 0.001 ‘***’, *p* < 0.01 ‘**’, *p* < 0.05 ‘*’). Frame refer to fish skeleton including fish fins.

*MPI* Metal pollution index.

Overall, the two fish species exhibited higher Al and Zn accumulation in all tissues, contributing to over 70% of MPI in all tissues However, differences were observed in the concentrations of HMs between slinger seabream and cape horse mackerel, depending on the specific organs examined. The observed variation in HM concentrations and MPI among the fish species investigated could be attributed to differences in their ecological requirements, metabolic processes, and feeding behaviors. Specifically, slinger seabream showed significantly higher concentrations of Al, As, Cr, Cu, Ni, and Zn in the liver followed by gills then muscle tissue (One-way ANOVA, *p* < 0.05, Table 1, 2). In addition, both gills and liver exhibited > twofold higher MPI values than muscles tissues (Table 1). These results are consistent with previous studies that show that fish liver stores and detoxifies trace elements, leading to higher accumulation levels compared muscle tissues (Coetzee et al. 2006; Plessl et al. 2019).

**Table 2:** One-way ANOVA of HM content in the various parts of cape horse mackerel and slinger-seabream

HM	Cape horse mackerel		Slinger-seabream	
	*F*_*(df*=*2)*_	*p*-value	*F*_*(df*=*2)*_	*p*-value
Al	13.68	< 0.0001***	13.78	< 0.0001***
As	9.94	0.0002***	11.95	< 0.0001***
Cr	3.223	0.0459*	3.905	0.0218*
Cu	2.151	0.1240	5.778	0.0037**
Mn	21.49	< 0.0001***	39.34	< 0.0001***
Ni	3.31	0.0424*	11.89	< 0.0001***
Pb	6.89	0.0019**	1.782	0.171
Zn	2.456	0.0933	33.12	< 0.0001***

*p < 0.05; ** *p* < 0.01; *** *p* < 0.001

Several studies have reported that factors such as dietary (carnivores/omnivore, dietary/prey preferences, trophic level), fish size, time of exposure (age), fish physiology (storage or elimination) and fish species lifestyle (whether migratory, dermersal, pelagic or benthic) may play an important role in the observed species HMs variability, even in those inhabiting similar polluted environment (Mendoza-Carranza et al. 2016; Okoro et al. 2016; Orata and Birgen 2016). The cape horse mackerel is positioned at a mid-trophic level in the marine food web, exhibiting opportunistic feeding behavior primarily focused on zooplankton like copepods and krill, as well as small fish and squid depending on prey availability. Acting as both predator and prey, it serves as an important food source for larger predatory fish, marine mammals, and seabirds (Georgieva et al. 2019; Kadila et al. 2020). In contrast, the slinger seabream occupies a higher trophic position and displays carnivorous feeding habits, targeting various organisms including small fish, crustaceans, mollusks, and benthic invertebrates. This versatile predator plays a crucial role in energy and nutrient transfer within the ecosystem and is susceptible to the bioaccumulation of contaminants, such as HMs, due to its position in the food web (Usero et al. 2005; Angel et al. 2010; Ahmed et al. 2016; Jiang et al. 2018). In this study, cape horse mackerel had higher bioaccumulation of Al, As and Pb in the gut than in the frame and muscles (One-way ANOVA, *p* < 0.05, Table 1, 2), indicating the importance of diet in the bioaccumulation of these metals. Similarly, the MPI for the gut was 1.5 fold higher than that of frame and muscles, indicating the importance of diet in the bioaccumulation of HMs in the fish species.

Accumulating evidence from multiple studies has shown that sediment serves as a critical pathway for the uptake of metal contamination, playing a significant role in the accumulation of HMs in fish. Fish species that inhabit areas near the sediments of water bodies and consume humic substances and benthic invertebrates tend to accumulate and transfer HMs from the sediments to their bodies. This leads to benthic and benthopelagic fish usually having higher metal concentrations than demersal fish (Jiang et al. 2018; Lozano-Bilbao et al. 2020). In addition, a large number of studies have shown that the bioaccumulation of HM in fish muscle is significantly correlated with fish species (Jitar et al. 2013; Li et al. 2015; Debipersadh et al. 2018). Consistent with such findings, muscle tissues of benthopelagic cape horse mackerel exhibited higher HM levels (MPI = 5.62) than the demersal slinger seabream (MPI = 3.96). However, slinger seabream exhibited higher bioaccumulation of HM in the gut than other tissues, indicating the impact of diet on HM exposure. In contrast, cape horse mackerel exhibited approximately threefold higher HM accumulation in the gills and liver tissues than muscle tissues, implying the higher affinity for metal absorption from contaminated water and food for this species. It is plausible that the deviation in HM accumulation patterns observed in this study could be attributed to fish physiology or difference in exposure levels. Furthermore, factors such as fish size, age, and migratory behavior may also contribute to the variation in HM accumulation patterns among fish species (Mendoza-Carranza et al. 2016; Okoro et al. 2016; Orata and Birgen 2016). Nevertheless, the findings of this study highlights the importance of habitat, diet, and lifestyle in determining the interspecies variability of HM accumulation patterns in fish in the Durban Basin.

### Comparison of HMs Accumulation Patterns of Fish Species from Cape Vidal and Durban Basin

**Fig. 2:**
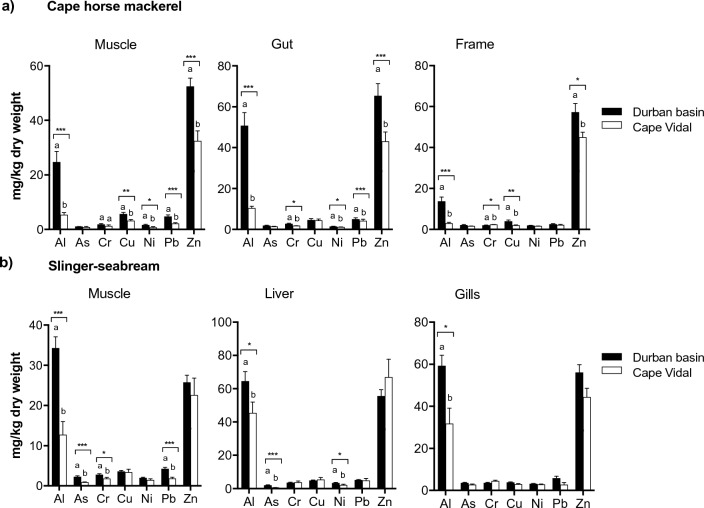
Comparison of the heavy metal accumulation pattern in different body parts of cape horse mackerel and slinger (*C. puniceus*) caught in the pristine (Cape Vidal) and polluted (Durban Basin) environment. Statistical differences at different *P* values are indicated (Mann–Whitney U test, *p* < 0.001 ‘***’, p < 0.01 ‘**’, p < 0.05 ‘*’)

This study also compared the trace element levels in two fish species, cape horse mackerel and slinger seabream, to determine their suitability as bioindicators of HM pollution in the two contrasting marine environments. We analyzed element concentrations in fleshy tissues, gut, and liver of the two fish species in Durban Basin (polluted habitat) and Cape Vidal (pristine ecosystem), and compared the trace element levels between the two fish species (Fig. 2).

In terms of individual metal and metalloid species, accumulation pattern of trace metals (mg/kg dm) in the edible muscle tissue of cape horse mackerel caught in Durban Basin followed the rank order Zn (52.5) >  > Al (24.7) >  > Cu (5.60) > Pb (4.72) > Cr (1.80 > Ni (1.64) > Mn (1.23) > As (1.04) (Table 1). Similar pattern was observed for cape horse mackerel caught in Cape Vidal; however, metal concentration values were lower than those reported in Durban Basin. Statistically, Mann–Whitney U test showed that the concentrations of Al (*p* < 0.001), Cu (*p* < 0.01), Ni (*p* < 0.001), Pb (*p* < 0.001), and Zn (*p* < 0.001) were significantly higher in cape horse mackerel samples caught in Durban Basin than in Cape Vidal. Consistent with MPI results, the Durban Basin cape horse mackerel samples had comparable Al, Cu, Ni, Pb and Zn levels that were 4.6-, 1.8-, 2.2-, 2.2-, and 1.6-fold higher, respectively, than Cape Vidal samples. With respect to slinger, metal concentrations (mg/kg dw) occurred in the rank order Al (34.2) > Zn (25.7) > Pb (4.25) > Cu (3.57) > Mn (3.41 > Cr > (2.75) > As (2.25 > Ni (1.96). Except for Cu, Ni and Zn concentrations that were statistically similar, the concentration of the remaining metals in slinger seabream fleshy tissue samples from Durban Basin and Cape Vidal were significantly different at *p* < 0.05 (Fig. 2). Intraspecific and regional differences in HM levels in both cape horse mackerel and slinger from the pristine (Cape Vidal) and polluted (Durban Basin) marine environments indicate their ability to bioaccumulate various kinds of pollutants at higher levels than the surrounding environment, and thus their potential use in pollution biomonitoring. Further, both species collectively exhibited higher accumulation of HMs levels in the liver (Al, Cr Cu and Zn) and gills (Mn and As) that were proportional to their environment concentrations. This implies that liver and gills HM levels in these two species are potentially good biomonitors of anthropogenic HMs water pollution.

Table 3 presents the metal concentrations in fish muscle samples from Cape horse mackerel and slinger seabream caught in Durban Basin and Cape Vidal, compared to maximum allowable limit (MAL) values and guidelines. Overall, the levels of Cu and Mn in the muscles of both fish species from the two study sites were below the maximum allowable levels (MAL) and guidelines values described in the literature. However, a significant cause of public concern emerged as the slinger seabream from Durban Basin had higher As, Cr, Ni and Pb levels than the MAL set by South Africa (DOH 2004), Australia/New Zealand (FSANZ 2013) and China (MHPRC 2013), European Commission (EC 2006) and Codex Alimentarius Commission (WHO/FAO 2015). In contrast, only Ni and Pb in slinger seabream caught in Cape Vidal were above the MAL. For cape horse mackerel, Durban Basin samples had higher levels of Ni, Pb and Zn above MAL, while Cape Vidal samples bioaccumulated Pb and Zn above the MAL (Table 3). Consistent with our findings, Moodley et al. (2021) recently reported a higher bioaccumulation of As, Cr and Pb in fish species (blacktail, karanteen, five finger, mullet, and pompano) caught in the South Durban Industrial Basin (SDIB), straddling the Isipingo Beach, Cuttings Beach, and Amanzimtoti Beach, South Africa. Similarly, we have also previously reported higher Pb and Zn of 8.44 and 56.71 μg g^−1^, respectively, in the fleshy tissues of cape horse mackerel in polluted Durban South (Debipersadh et al. 2018), than reported in the current study. These values were comparable to similar fish species caught in coastal water of Turkey (Mutlu et al. 2012), but lower than those caught in polluted coastal estuaries and habors (Angel et al. 2010; Vilches et al. 2019).

**Table 3:** The mean (± SE) concentrations of heavy metals and metal pollution index (MPI) measured in water, sediments, muscles of slinger seabream and cape horse mackerel caught in the Durban Basin and maximum permissible limits for fish (mg/kg dw)

Species/Location	Al	As	Cr	Cu	Ni	Pb	Zn	MPI
*Sediments (mg/kg)*								
Durban Basin	1437 ± 121	1.4 ± 0.17	12 ± 2.61	2.8 ± 0.62	3.6 ± 0.83	2.0 ± 0.31	12 ± 1.83	8.40
Cape Vidal	1338 ± 234	0.8 ± 0.11	6.9 ± 0.83	1.4 ± 0.45	3.0 ± 0.96	1.0 ± 0.19	3.2 ± 0.99	5.18
*Seawater*								
Durban Basin	39 ± 3.8	3.9 ± 0.1	1.4 ± 0.05	3.9 ± 0.79	0.8 ± 0.16	2.8 ± 0.49	17 ± 4.67	4.40
Cape Vidal	34 ± 2.1	3.6 ± 0.32	1.1 ± 0.00	1.3 ± 0.09	0.3 ± 0.01	0.4 ± 0.12	1.2 ± 0.77	1.59
*Slinger-seabream*								
Durban Basin	34 ± 2.9*a*	**2.3 ± 0.2*a***	**2.6 ± 0.2*a***	3.6 ± 0.2	**2.0 ± 0.1*a***	**4.1 ± 0.3*a***	30 ± 2.0a	5.64
Cape Vidal	13 ± 3.3*b*	0.9 ± 0.1*b*	1.8 ± 0.3*b*	3.4 ± 0.4	**1.5 ± 0.3*b***	**1.8 ± 0.3*b***	23 ± 4.3*b*	3.32
*P*-value	< 0.0001	0.0023	0.0413	0.9781	0.0287	0.0013	0.0134	
*Cape horse mackerel*								
Durban Basin	25 ± 3.9	1.0 ± 0.1	1.7 ± 0.3	5.6 ± 0.6	**1.6 ± 0.2**	**4.7 ± 0.6**	**52 ± 3.01**	5.13
Cape Vidal	5.4 ± 0.9	0.8 ± 0.3	1.3 ± 0.5	3.2 ± 0.4	0.7 ± 0.3	**2.2 ± 0.3**	**32 ± 3.7**	2.64
*P*-value	< 0.0001	0.5671	0.0831	0.0301	0.0039	**0.0058**	**0.0134**	
*Maximum permissible limits*								
FAO (FAO 1983)	–	–	–	30	–	–	30	
EC (EC 2006)	–	–	–	–	–	0.3	–	
Codex Alimentarius (WHO/FAO 2015)	–	1.4	–	30	–	0.3	100	
China (MHPRC 2013)	–	0.1	2	–	–	0.5	–	
Australia and New Zealand (FSANZ 2013)	–	2	–	–	–	0.5	–	
South Africa (DOH 2004)	–	3	–	–	–	0.5	–	
US (Institute of Medicine 2001)	–	–	–	10	1	–	40	

^#^Values higher than maximum allowable limits (MAL) are shown in bold

Pb is known to possess various adverse effects such as neuro- and nephrotoxicity, rapid behavioral malfunction, and decrease in the growth, metabolism, and survival rate, alteration of social behavior in some mammals (Damiano et al. 2011; Bosch et al. 2016; Fakhri et al. 2018). In contrast, As does not have a recognized biological function and typically accumulates in aquatic organisms through direct uptake from water, usually occurring only at elevated environmental concentrations. The toxicity of As not only depends on its concentration, but also on its speciation (Jain and Ali 2000), with the inorganic As being highly carcinogenic (Pei et al. 2019). The total As in marine fish reported within a range of 0.2–150 μg g^−1^ while bioaccumulation varies for different tissues (Pei et al. 2019). At very low concentrations, Cr play an important role in regulating lipid and glucose metabolism, but excessive intake can have adverse effects on pulmonary health, and may result in damage to organs, such as the lungs, kidneys, and liver (Vu et al. 2017). The elevated levels of Pb, As and Cr recorded in all fish species in this study therefore warrant concern due to potential public health and environmental impacts.

The MPI values reported in this study were, however, lower than those reported for fresh water fish species such as *Carassius auratus* (0.059), *Pelteobagrus fulvidraco* (0.073) and *Hypophthalmichthys nobilis* (0.046) from Nansi Lake, China (Li et al. 2015). Similarly, Chi et al. (2007) have reported MPI values between 0.2 and 0.7 for *Cyprinus carpio*, *Carassius auratus*, *Hypophthalmichthys molitrix* and *Aristichthys nobilis* from Taihu Lake, China. However, marine fishes have reported higher MPI values than fresh water ecosystem, with the values being significantly higher in polluted marine and estuarine environments (Ali and Khan 2018). Higher MPI values for *C. puntatus* exposed to thermal power plant effluent have been reported for gills (53.6), kidneys (41.2), integument (34.2), liver (31.9) and muscles (13.5) (Javed et al. 2016). These values are higher than those reported in this study, which is partly attributed to the dilution effect of the pollutants by seawater. Consistent with our findings, Rios-Fuster et al., (2019) also reported higher HMs accumulation in *T. mediterraneus*, a close relative to the cape horse mackerel (*T. trachurus*), whereas time-dependent accumulation of Zn and Pb in *T. trachurus* in polluted environments have also been reported (Jitar et al. 2013; Lozano-Bilbao et al. 2020), indicating potential as biomonitor for metal pollution. To the best of our knowledge, this study is the first report on higher HMs accumulation in slinger seabream (*C. puniceus*) compared to other species making it a promising candidate as bioindicator for pollution in the Durban coast. Furthermore, the different patterns in both species collectively suggest that the fish muscle is not an active tissue in accumulating HMs, whereas liver and gills are good biomonitors of water pollution with metals, as their concentrations were proportional to those present in the environment.

Overall, the study has shown that cape horse mackerel and slinger seabream can be useful biomonitoring species for detecting moderate levels of HM pollution in marine ecosystems and differences between geographical locations. However, it is important to note that species-specific differences in metal levels, as discussed above, may not be efficient for monitoring small differences such as time trends without a more detailed analysis of the monitoring data. Therefore, future studies should consider comparing time trends in heavy metal accumulation patterns in both water and fish species in the two geographical locations to fully demonstrate the indicator capabilities of cape horse mackerel and slinger seabream. In addition, further biomonitoring studies that evaluate native species responses and their ability to indicate environmental degradation are necessary to gain a more comprehensive understanding of the impacts of heavy metal pollution on marine ecosystems.

### Human Health Risk Assessment

**Fig. 3:**
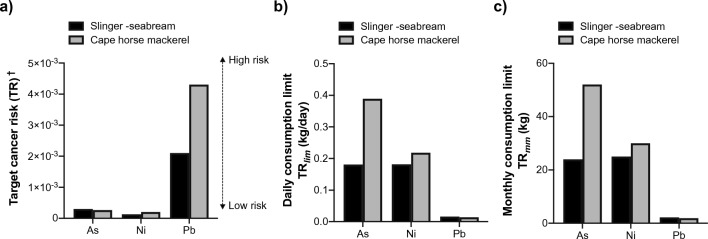
Carcinogenic health risk of toxic heavy metals via consumption of slinger seabream and cape horse mackerel caught in the Durban Basin. **a** Lifetime target cancer risk (TR) ^−^ used to indicate the carcinogenic risk (lifetime TR > 10^−4^, unacceptable; TR = 10^−6^ ~ 10^−4^, an acceptable range; and TR < 10^−6^, negligible (Ahmed et al. 2016)). **b** Daily (*TR*_*lim*_) and maximum allowable and **c** monthly consumption limit (TR_mm_) for As, Ni and Pb relating to the number meals of two fish that can safely be eaten per month with no adverse carcinogenic health effects

Considering the substantial consumption of fish in Durban Basin particularly the edible muscle part, it is important to acknowledge the potential risks associated with fish intake among the local population. The risk assessment results are summarized in Table 4 and Fig. 3 for EDI (estimated daily intake), THQ (target hazard quotient), and CR (carcinogenic risk), respectively.

**Table 4:** The estimated daily intake (EDI), maximum daily intake (MDI) and target hazard quotient (THQ) of heavy metals via consumption of slinger seabream and cape horse mackerel in the Durban Basin by adult person

Indices	Heavy metal							HI^∂^ (TTHQ)
	Al	As	Cr	Cu	Ni	Pb	Zn	
*EDI (µg/day/person)*								
Slinger-seabream	12.6	0.81	0.99	1.26	0.71	1.53	9.28	
Cape horse mackerel	8.90	0.38	0.65	2.02	0.59	1.71	8.9	
TDI^1^ (µg/kg body weight/day)	143	2.14	0.3	500	5	1.2	1000	
PTDI_60.7_^2^	8680.1	129.9	18.21	30,350	303.5	216.7	60,700	
*MDI*^*3*^* (g)*								
Slinger-seabream	249	58	6.6	8501	155	51	2362	
Cape horse mackerel	352	125	10	5419	185	46	1158	
*THQ*								
Slinger-seabream	0.089	0.378	3.310	0.003	0.142	0.430	0.009	4.370
Cape horse mackerel	0.062	0.177	2.169	0.004	0.118	0.478	0.019	3.029

^1^TDI: tolerable daily intake (µg/kg body weight/day), calculated from tolerable weekly intake (TWI) cited in Türkmen et al. (2009) after FAO/WHO (2004); TWI for Cr (EFSA 2014)

^2^PTDI_60.7_: permissible tolerable daily intake for a 60.7 kg person (µg/day) = TDI × 60.7 kg

^3^Values between brackets are the maximum daily intake (in g) of each fish species that should be consumed in order to attain the permissible tolerable daily intake of metal for 60.7 kg person (= PTDI_60.7_ (µg/day)/metal concentration (µg/g) according to FAO/WHO (2004)

^#^THQ – is the non-carcinogenic risk and is dimensionless; (THQ > HI = 1, indicate potential health risk of consuming the specific metal through fish (Varol and Sünbül 2017)

^∂^HI (or TTHQ) – is the sum of the target hazard quotient; (HI > 1 indicates potential health risk all the metal species through the fish consumption (Varol and Sünbül 2017))

#### Estimated Daily Intake (EDI)

Based on human-biomonitoring data, the risk assessment of different HMs based on indices such as estimated daily intake (EDI) and tolerable daily intake (TDI) can be individually evaluated (Table 4). While EDI can be presumed as to be the daily consumption of a HM residue, TDI refer to the permissible intake amount that can be ingested orally daily over a lifetime without an appreciable health risk, whose values have been determined for most HMs and other pollutants (Varol & Sünbül, 2017; Nordberg & Fowler 2019). In South Africa, average annual fish consumption per capita per person is estimated at 6–8 kg (~ 21.92 g day^−1^) (Grünberger 2014). Therefore, daily intake of HMs was estimated on the basis of the concentrations measured in fish muscle and daily fish consumption rate (21.92 g), and the metal intakes compared with the respective permissible tolerable daily intake for South African adult average body mass 60.7 kg (PTDI_60.7_) (μg day^−1^). Among the metals studied, EDI for Cr in all the fish species was higher than permissible TDI for an adult, implying potential health risk associated with the intake Cr through fish consumption.

#### Non-carcinogenic Health Risk (NCHR) Assessment (THQ and HI)

Table 4 also presents the target hazard quotients (THQs) and hazard index (HI or TTHQ) of the metals studied in two fish species samples from Durban Basin. A THQ value below 1 is considered low, indicating that the exposed population is unlikely to experience adverse effects (Wang et al. 2005). The study found out that only Cr had THQ > 1 in all the fish species, with highest value for slinger seabream (3.31) followed by cape horse mackerel (2.17). This indicates that Cr may pose a significant non-carcinogenic health hazard to human beings consuming these two fish species in the Durban Basin. Furthermore, all the fish species had hazard index (HI) value above 1, indicating a significant non-carcinogenic health risk to adults in Durban Basin due to the intake of either Cr or the six metals present in the two commonly consumed edible and baitfish species under study. Consistent with THQ results, slinger seabream had comparatively higher HI value than cape horse sea mackerel. The findings that the hazard index (HI) values for each fish (obtained by summing up the THQ of all the metals detected and quantified in each fish) were greater than 1, further confirming that the potential health and food security concerns arising from metal bioaccumulation in the assessed fish species is, at the present stage, significant (Varol & Sünbül, 2017).

#### Carcinogenic Risk (CR) Assessment

In order to evaluate the potential carcinogenic risk associated with the consumption of fish containing HMs, this study calculated the target carcinogenic risks (TR) for As, Ni and Pb, which are known to promote both non-carcinogenic and carcinogenic effects depending on the exposure level. The TR values were estimated using the cancer slope factor (CSF) of 1.5, 1.7 and 0.009 mg/kg/day for As, Ni and Pb, respectively, as recommended by USEPA (USEPA 2016). The results are presented in Fig. 3.

Results showed that the TR values for As were 3.0 × 10^−4^ and 2.6 × 10^−4^ for slinger seabream and cape horse mackerel, respectively. Consistent with our findings, Moodley et al. (2021) also reported a TR value < 10^–3^ for As, and proposed that fish from the SDIB should be consumed in moderation or not consumed to prevent long-term toxic effects of As. For Ni, the TR values were 1.3 × 10^−4^ and 2.0 × 10^−4^ for slinger seabream and cape horse mackerel, respectively. However, the TR values for Pb in slinger and cape horse mackerel were < 10^–4^, indicating a potential cancer risk associated with the consumption of these fish species from the Durban Basin.

Figure 3 also illustrates the results of daily consumption limits (*TR*_*lim*_) and the maximum allowable monthly consumption limit (TR_mm_) for As, Ni, and Pb in relation to the number of fish meals that can be safely consumed per month without causing adverse carcinogenic health effects. For As and Ni, the TR_lim_ ranges between 181 and 389 and 182–361 g/day, respectively, which are higher than the average adult fish consumption in South Africa (Fig. 3b). The results indicate that an adult can safely consume 24 and 52 meals/month of slinger seabream, and cape horse mackerel, respectively, without experiencing any negative cancer health effects due to As exposure. Similarly, between 25 and 30 meals/month of these fish species can be safely consumed without any cancer health effects due to Ni exposure. In contrast, the maximum allowable daily consumption limits (TR_lim_) for Pb in these two fish species were 16 and 14 g/day, which is lower than the daily fish consumption rate by adult South Africans (21.9 g/day). These results indicate potential health safety concerns for consumers who consume these fish species caught in the Durban Basin. Furthermore, the calculated TR_mm_ values were lower than the recommended safe fish consumption rate of > 16 meals/month by USEPA (USEPA 2000). Perhaps the most significant finding of the study was the almost twofold higher levels of As, Cr and Zn in slinger than cape horse mackerel in the same location, that were also above the MAL. Based on these results, an advisory on limited consumption of slinger seabream from the Durban Basin is needed.

### Relationship of HMs in the Fish Muscle Tissues

**Fig. 4:**
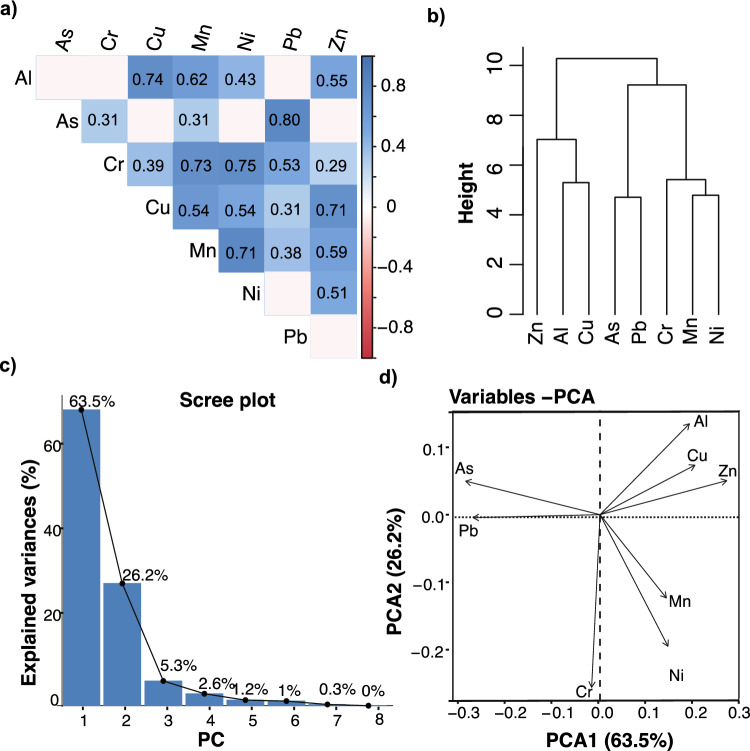
Relationship of HMs in the fish muscle tissues **a** Correlogram of heavy of heavy metals in the body tissues in different body parts of cape horse mackerel and slinger seabream in Durban Basin. **b** Hierarchical cluster analysis (dendrogram) of the variables (metals) in the study area. **c** Scree plot showing the contribution of principal components (PC) to the variability of HMs in the fish muscles in the study site. **d** Loadings plot of rotated PCA of 7 metals in the fish sample

In this study, we utilized a combination of Pearson correlation and hierarchical clustering based on Euclidean distance to assess the correlation between HMs in fish muscle tissues and their potential sources. Figure 4a illustrates the Pearson correlation matrix among the HMs in the fish muscle tissue in the Durban Basin. The correlation matrix revealed significant strong positive correlations (ranging from 0.71 to 0.80 at *P* < 0.05) among various HMs, such as Al and Cu, As and Pb, Cr and Mn, Cr and Ni, Mn and Ni, and Cu and Zn.

Statistically significant HMs with high correlation coefficients are considered to have the same origin or similar behavior in the marine environment (Angel et al. 2010). These findings were further supported by cluster analysis, which grouped the HMs into homogenous clusters based on similarities within the same group and dissimilarities between different groups (Fig. 4b). Cluster 1 comprised Al, Zn, and Cu, which could be attributed to natural sources such as rock and soil weathering, as well as human activities such as chemical and pharmaceutical industries, and industrial effluents, among others. On the other hand, Cluster 2 included As, Pb, Cr, Mn, and Ni, which may be linked to surface runoff from agricultural activities, shipping activities, and municipal and industrial wastewater. This pattern of HM distribution in marine environments is consistent with previous studies conducted in other regions around the world, including East London and Port Elizabeth harbor in Eastern Cape Province, Cape Town harbour in South Africa, and other locations with similar economic activities such as shipping, petrochemical, and automotive industries (Angel et al. 2010; Okoro et al. 2016; Jupp et al. 2017; Lao et al. 2019).

In this study, we also employed principal component analysis (PCA) to extract significant principal components (PCs) and their associated loads in order to further investigate the internal correlation of variables and trace the sources of HMs pollution. The results of PCA, as depicted in Fig. 4c and d, revealed that the first principal component (PC1) explained 63.5% of the variance, the second principal component explained 26.2%, and the combined three principal component axes explained 95.0% of the overall variance (Fig. 4c). Similar to hierarchical clustering (Fig. 4b), the vectors of Al, Zn, and Cu clustered separately from the other HMs. As and Pb also clustered together, distinct from Cr, Mn, and Ni, indicating different degrees of loading on the principal components. Specifically, on PC1, Mn, Cu, Cr, and Al contributed to high loadings (> 0.7), while As and Pb were the only metal species with positive loadings (> 0.7) on PC2. However, Al, Zn, and Cu exhibited negative loadings on PC2 (< 0.2976). These results further support the conclusion that the sources of these three elements are significantly different from the other HMs in the Durban Basin, particularly As and Pb. In addition to common sources of HM pollution, such as storm water drains and streams carrying runoff from industrial, urban, and residential areas, the Durban harbor may also be impacted by ship repair activities, antifouling paint rich in copper, oil spills from boats, and coal handling activities in the docks, which could contribute to the observed higher levels of HMs such as Cu, Pb, and Zn in the area. Furthermore, a review of studies on As in African waters indicates elevated concentrations in both surface water and groundwater, primarily attributed to mining operations, agricultural drains, local sediments, disposal, and municipal and industrial wastes (Ahoulé et al. 2015). Collectively, these findings highlight the urgent need for continuous monitoring of pollutant levels in various components of marine environments to effectively manage and mitigate HM pollution.

## Conclusion

In summary, this study examined the presence of HM in cape horse mackerel and slinger seabream from the Durban Basin in South Africa. The analysis found that Zn and Al were the most prevalent HMs, constituting over 70% of the MPI. Slinger seabream showed higher concentrations of HMs in their liver and gills, while cape horse mackerel had higher levels in their gut. The accumulation of HMs in fish was influenced by factors, such as diet, habitat, and lifestyle. A comparison of fish species from Cape Vidal and the Durban Basin highlighted the impact of habitat, diet, and lifestyle on HM accumulation. These two fish species should be used as bioindicators to assess the extent and severity of HM pollution in the marine environment. The study also conducted a human health risk assessment, which revealed potential HM contamination in fish from the Durban Basin. Both the EDI and THQ for Cr exceeded PTDI, suggesting a health risk for adults. Specifically, THQ values above 1 for Cr for the two fish species indicated potential significant non-carcinogenic health hazards from their consumption. The HI, calculated by summing THQ values for all metals, exceeded 1, indicating substantial health risks from metal bioaccumulation. The two fish species also showed potential carcinogenic risks for Pb, with TR values surpassing acceptable levels (10^–4^). These findings highlight the importance of limiting the consumption of fish from the Durban Basin to reduce long-term exposure to HMs. It is essential to note that while this study focused on the Durban Basin, caution should be exercised when generalizing these findings to all fish along KwaZulu Natal's Indian Ocean coastline due to the region's diverse marine biodiversity. Additionally, it is crucial to consider official guidelines and recommendations provided by recognized health and regulatory authorities when assessing the implications of these findings. Nevertheless, the consistently high levels of anthropogenic HMs in fish species, surpassing regulatory limits for seafood, emphasize the urgent need for comprehensive national and regional strategies to address and manage HM pollution in the Durban Basin.

To further enhance our understanding of the accumulation of harmful metals (HMs) and their impact on Durban Basin fish, human health, marine health, and environmental health, this study has identified several areas that require further research. Firstly, future investigations should aim to provide more precise determination of food sources, trophic position in food webs, and the progressive time-integrated enrichment and transfer pathways of HMs. Stable isotope ratios of carbon (^13^C:^12^C; δ^13^C) and nitrogen (^15^N:^14^N; δ^15^N) can be utilized for this purpose with a focus on metals and metalloids known for their persistence and biomagnification, such as MeHg, Cd, and Se. Intrinsic species factors, including fish size, age, physiology (storage or elimination), and lifestyle (demersal, pelagic, or benthic), should also be considered to better understand metal accumulation patterns. Secondly, studies investigating the movements and dispersal patterns of fish species within the Durban coastline and other waters are necessary. Further research should also examine location-specific environmental and biological parameters such as water depth, water and sediment temperature, organic matter levels in the water column and sediments, and identification of metal input sources that may affect the bioavailability of metals. Lastly, it will also better to understand the influence of metal levels in the species studied and their overall ecosystem health and human health implications.

## Data Availability

All data generated or analyzed during this study are included within the article.
